# Growth, Hemato-Biochemical Parameters, Body Composition, and Myostatin Gene Expression of *Clarias gariepinus* Fed by Replacing Fishmeal with Plant Protein

**DOI:** 10.3390/ani11030889

**Published:** 2021-03-20

**Authors:** Mohammed A. F. Nasr, Rasha M. Reda, Tamer Ahmed Ismail, Amira Moustafa

**Affiliations:** 1 Department of Animal Wealth Development, Faculty of Veterinary Medicine, Zagazig University, El-Zeraa Street. 114, Zagazig 44511, Egypt; nasr.maf@gmail.com; 2 Department of Fish Diseases and Management, Faculty of Veterinary Medicine, Zagazig University, Zagazig 44511, Egypt; 3 Department of Clinical Laboratory Sciences, Turabah University College, Taif University, P.O. Box 11099, Taif 21944, Saudi Arabia; t.ismail@tu.edu.sa; 4 Department of Physiology, Faculty of Veterinary Medicine, Zagazig University, Zagazig 44519, Egypt; amiramostafa@zu.edu.eg

**Keywords:** *Clarias gariepinus*, body composition, economic profit growth parameters, plant protein

## Abstract

**Simple Summary:**

The costs of feed ingredients in the aquaculture sector are one of the main problems impacting the success or failure of a business. The key ingredient in aquafeeds and the costliest among them is fish meal (FM). Therefore, great consideration was given to the use of different types of plant protein (PP) meals in aquafeeds (soybean and sunflower meal). In this study, fish were divided into five groups, with each group in triplicate (30 fish/group; 10 fish/replicate). Group 1 was fed the control diet consisting of 15% FM and 41% soybean meal. The other four groups (D1, D2, D3, and D4) were fed experimental diets, where FM was replaced gradually by plant protein sources (33, 50, 66, and 100% soybean meal and sunflower meal) for 60 days. In conclusion, partial or total replacement of FM with a plant protein source (soybean and sunflower meal) showed similar growth performance and body composition with greater economic efficiency.

**Abstract:**

In this study, we evaluated the consequences of replacement of fishmeal with plant protein sources (soybean and sunflower meal) on fish growth parameters, haemato-biochemical factors, body composition, and myostatin gene expression of *Clarias gariepinus*. A total of 150 *C. gariepinus* were organized in glass aquaria into five investigational groups, with each group in triplicate (30 fish/group; 10 fish/replicate). Group 1 was fed a control diet (15% fishmeal). The other groups were fed diets where fishmeal was replaced gradually with plant protein, with 10% fishmeal in the second group (D1), 7.5% fishmeal in the third group (D2), 5% fishmeal in the fourth group (D3), and 0% fishmeal in the fifth group (D4). There were no significant differences regarding growth performances and body composition among the groups, except that the feed conversion ratio was improved in D4. The different diet types did not affect hematologic parameters and blood indices. Serum growth hormone and amylase levels also revealed no significant (*p* = 0.09 and 0.55, respectively) differences among the groups, while serum lipase levels decreased significantly (*p* = 0.000) due to partial (D2) or complete (D4) substitution of fishmeal with plant protein. The replacement of fishmeal had no effects on liver (*p* = 0.51) and kidney functions (*p* = 0.34). However, D4 showed the best profit and economic efficiency compared to the other groups. Altogether, we concluded that substitution of fishmeal with plant protein sources is economically beneficial and may be without any adverse effects on growth parameters, body composition, or hematologic and biochemical parameters, but with the addition of synthetic amino acids.

## 1. Introduction

Fish and fish products have a significant role in food security and nutritional needs of the human population in developing and developed countries [1]. With the amino acid content of fish protein, it is of superior biological value, comparable to that of milk, eggs, and beef. Additionally, these products (fish and fish products) contain high-quality protein, vitamins, minerals, and polyunsaturated omega-3 fatty acids, and are easily digested [2,3,4]. The muscles have extra edible lean tissue as compared to beef, pork, and poultry [5].

The African catfish, *Clarias gariepinus*, is the foremost warm water aquaculture species in Africa, Asia, and recently Europe and Latin America [6]. *C. gariepinus* should be considered and reared more, especially in Africa, because of its good conversion rates and fast growth despite being fed a low-quality diet and its high stocking density [7]. With *C. gariepinus* being characterized by disease and stress resistance [8], these features account for its global commercial importance [9]. 

Fish feed contributes approximately 40% to 60% of production costs in aquaculture [10]. Fishmeal (FM) is the principal component in feeding fish that are characterized by palatable protein, and for decades, it has been the main protein source in fish feed due to its high-quality protein content, amino acids, vitamins, minerals, and further unidentified growth factors [11,12]. Owing to the fast growth in fish farming, FM prices have increased in the past decade and are expected to increase further to meet the sustained growth [13]. Therefore, plant protein (PP) sources are being considered as an alternative source for protein in diet formulations as a partial or complete replacement for FM [14,15] for several reasons, such as their inexpensive cost and more sustainable source [16]. Consequently, the main concern of recent investigations is the reduction and/or potential elimination of FM use in fish diet formulation [16]. 

Soybean meal is the finest component to replace FM [17]. It is readily available and rich in high quality protein compared to other plant protein feedstuffs [17,18]. As a derivative of sunflower (*Helianthus annuus*) seed oil extraction, sunflower meal is considered as an alternative protein supply for fish feed production [19]. It has been used for fish nutrition due to its higher contents of sulfur amino acids and low anti-nutritional factors, except for tannin and phytic acid, compared to other oilseed meals [20,21,22]. 

Partial replacement of fishmeal with sunflower meal in Trout feed revealed significant success and better growth rate [23]. Moreover, it could be decreasing the cost without affecting growth performance [24]. Additionally, Rahmdel et al. [25] stated that sunflower meal could replace up to 75% of fishmeal without any negative effect on growth performance, body composition, and haemato-biochemical indices for common carp fingerlings. These results were supported by Nogales Merida, Jover Cerda, Martínez-Llorens, and Tomás Vidal [22], who detected that there was no significant effect of replacing fishmeal with different levels of sunflower meal for sharp snout sea bream, *Diplodus puntazzo* (Walbaum). On the other hand, Olvera-Novoa, Olivera-Castillo, and Martínez-Palacios [21] detected a reduction of growth performance of tilapia, *Tilapia rendalli* (Boulenger), after replacing fishmeal with more than 20% sunflower meal. To our knowledge, this is the first study to explore the effects of partial or total replacement of FM with soybean and sunflower meal on fish growth performance, haemato-biochemical parameters, body composition, and myostatin gene expression of *C. gariepinus*.

## 2. Materials and Methods 

### 2.1. Ethical Statement

The experimental procedure was approved by the Ethics of the Institutional Animal Care and Use Committee of Zagazig University, Egypt (ZU-IACUC/2/F/139/2020). 

### 2.2. Experimental Diet

Five experimental diets were formulated using commercial ingredients to fulfill the nutrient requirements of *C. gariepinus* according to the National Research Council (NRC) [26] and Hepher et al. [27] (Table 1). The control diet (commercial diet) consisted of 15% FM. The other four groups were fed experimental diets, where fishmeal was replaced gradually with plant protein 10% fish meal in the second group (D1), 7.5% fish meal in the third group (D2), 5% fish meal in the fourth group (D3), and 0% fish meal in the fifth group (D4). All experimental diets were formulated to be isonitrogenous (the level of crude protein was constant) and isocaloric. By replacing FM with plant protein sources, the different diets were supplemented with: (1) synthetic amino acids, such as methionine and lysine, to maintain constant levels of those amino acids; and (2) dicalcium phosphate to cover available phosphorus requirements, because FM is rich in essential amino acids and phosphors. The diets were manufactured at the Fish Research Unit, Faculty of Veterinary Medicine, Zagazig University, and were pelleted in a meat mincer to 3 mm, air-dried, and then stored at 4 °C throughout the feeding period. A representative sample from the feed of each group was submitted for proximate chemical analysis, according to the methods of the AOAC [28], while the amino acid contents were analyzed according to the method of Llames and Fontaine [29].

### 2.3. Fish, Experimental Condition, and Feeding Schedule

In total, 150 *C. gariepinus* (51.01 ± 0.34 g) were collected from a private fish farm, Sharkia Province, Egypt. Fish were transported in a disinfected, well-constructed tank with an efficient aeration system early in the morning, to avoid heat and sunshine, to the Department of Fish Diseases and Management, Faculty of Veterinary Medicine, Zagazig University, Egypt. The water in the transport tank was gradually replaced with the source of water in the lab for acclimatization to lab temperature and water quality conditions. The fish was held for two weeks to acclimatize to the condition of the laboratory environment and during this time fish was fed on control diet (15% fishmeal). Fish were distributed randomly in glass aquaria (80 × 40 × 30 cm, water capacity =60 L) into five groups, with each group in triplicate (30 fish/group; 10 fish/replicate). Group 1 was fed the control diet that consisting of 15% FM and 41% soybean meal. The other four groups (D1, D2, D3, and D4) were fed experimental diets, where FM was replaced gradually by plant protein sources (33, 50, 66, and 100% soybean meal and sunflower meal) for 60 days. Group 2 (D1) received 10 and 50.3% (45.3% soybean and 5% sunflower meal), Group 3 (D2) received 7.5 and 54.6% (47.5% soybean and 7.1% sunflower meal), Group 4 (D3) received 5 and 59% (50.9% soybean and 8.1% sunflower meal), and group 5 (D4) received 0 and 60% (50% soybean and 10% sunflower meal) of fishmeal and plant protein, respectively. Fish were fed at a rate of 3% total fish biomass manually by hand twice per day (9 a.m. and 2 p.m.). The amount of food was adjusted every two weeks according to the change in fish body weight. Water parameters were kept at standard values [30] throughout the experimental period (temperature, 26.5 ± 0.5 °C; pH, 6.2 ± 0.4; ammonia, 0.025 ± 0.003 mg/L and nitrite, 0.015 ± 0.002 mg/L). Water was exchanged daily at a rate of 30%.

### 2.4. Sampling and Analytical Methods

#### 2.4.1. Growth Parameters and Morphological Index

The growth index was evaluated by the final body weight (FBW), weight gain (WG), specific growth rate (SGR), feed conversion ratio (FCR), viscero-somatic index (VSI), hepato-somatic index (HSI), and intestinal body ratio according to the following formulas:

WG (g) = final weight − initial weight.

SGR%= [(Ln final weight − Ln initial weight)/60 days] × 100.

FCR = feed intake (g)/weight gain (g).

VSI% = viscera weight × 100/body weight.

HSI% = hepatopancreas weight × 100/body weight.

Intestinal body ratio = [intestinal length (cm) body length (cm)] × 100.

#### 2.4.2. Hematologic Analysis

At the end of the experiment (60 d), nine fish/group (three fish/replicate) were collected and anesthetized with 100 mgL^−1^ benzocaine solution (Al-Nasr pharmaceutical chemicals Co, Oubour, Qalyubia, Egypt) [31]. The blood samples were collected by using 1 mL syringes that rinsed with EDTA solution (10%; it was used as 1:5 volumes of blood) [32] from caudal blood vessels. The hematologic indices, including red blood cell counts (RBCs), hematocrit (Hct%), hemoglobin concentration (Hb), mean corpuscular volume (MCV), mean corpuscular hemoglobin (MCH), and mean corpuscular hemoglobin concentration (MCHC), were determined using a Sysmex XT-2000iV Automated Hematology Analyzer (Sysmex Corporation, Hyogo, Japan) at the Animal Health Research Institute, Zagazig Branch, Egypt.

#### 2.4.3. Biochemical Analysis, Serum Growth Hormone (GH), and Digestive Enzymes

Blood was collected into clean glass tubes for serum separation at the end of the experiment (three fish/replicate; nine fish/group) and allowed to clot at room temperature for 2 h. The blood was then centrifuged for 15 min, at 3000 rpm. A rapid colorimetric kit (BioAssay Systems, Hayward, CA, USA) was used to evaluate serum amylase and lipase digestive enzymes. Serum GH, alanine aminotransferase (ALT), aspartate aminotransferase (AST), urea, creatinine, and blood urea nitrogen (BUN) were determined with commercial kits (Wako Pure Chemical Industries, Osaka, Japan).

#### 2.4.4. Body Composition Chemical Analysis

Whole fish body composition chemical analysis was evaluated after 60 days of feeding following AOAC [28]. Crude protein (Kjeldahl method), total lipids [33], and ash contents [28] were evaluated.

#### 2.4.5. Liver Histology and Intestinal Morphometric Analysis

Specimens from the fish liver and intestine (three fish/replicate; nine fish/group) were collected and fixed immediately in 10% buffered neutral formalin solution for 48 h, dehydrated in gradual ascending ethanol (70, 80, 95, 95, and 100%), cleared in xylene and embedded in paraffin. Five-micron thick paraffin sliced using a microtome (RM 2155; Leica, London, UK). The sections were prepared, routinely stained with hematoxylin and eosin stains (H&E) and examined microscopically [34]. Morphometric analysis was done with camera microscope AmScope^®^ software as villus height measured (µm) from the tip to the base of the villus and diameter. Submucosa layer thickness, number of goblet cells per area of epithelium layer, intraepithelial leucocytes, and lamina propria leucocytes were also calculated. 

#### 2.4.6. Myostatin Gene Expression

Spleen tissue samples [35,36] were collected from three fish/replicate; nine fish/group (from the same dissected fish samples to obtain liver and intestine samples for histology and morphometric analysis) at the end of the feeding period for total RNA extraction according to the manufacturer’s protocol using the QIAamp RNeasy Mini kit (Qiagen, Hilden, Germany, GmbH). Complementary DNA was produced following manufacturer’s instructions (Quantitect^®^ Reverse Transcription kit, Qiagen). Quantitative real-time PCR analysis was performed with SYBR green PCR master mix (Step One Plus, Applied Biosystem, Foster City, CA, USA). EF-1α (Genbank accession number = AB075952.1) (Forward: 5′-CCTTCAACGCTCAGGTCATC-3′; Reverse: 5′-TGTGGGCAGTGTGGCAATC-3′) was chosen as internal standard as suggested by Gröner et al. [37]. The target gene was myostatin (*MSTN*, Genbank accession number = KJ372760.1) (Forward: 5′-CAACGATCTGGCTATCACTTCTGC-3′; Reverse: 5′-CGAGCAGTAGTTAGCTTTGTAGCG-3′) [38]. The amplification conditions were 40 cycles (94 °C for 15 s, 58 °C for 30 s, and 72 °C for 30 s). The amplification efficiency of the primer was determined by standard curve assay. Amplification efficiencies were >97% for each group. 

#### 2.4.7. Economic Efficacy ($)

The economic efficiency was calculated by the following formulas:

Feed cost/kg body weight gain (BWG) = FCR × cost of 1 kg diet.

Profit/kg weight gain = Return/kg gain (price of kg) − Feed cost/kg gain

Economic efficiency = Profit/feed cost per kg weight gain

### 2.5. Statistical Analysis

Statistical analyses were performed using the SAS statistical system package (SAS 2008; SAS Institute, Cary, NC, USA). The Kolmogorov-Smirnov test was applied to verify the normality of values. Homogeneity of variance was verified with Levene’s test. A one-way analysis of variance used to detect variations among the groups. Tukey’s test was used to verify the presence of significant differences among the treatments at a level of 5%. The replicates did not present a significant effect; therefore, the results from the three replicates were combined.

## 3. Results

### 3.1. Growth Performances and Biometric Indices

Fish in all experimental groups accepted the experimental diets with a 100% survival rate during the 60 days of feeding. There was no significant effect of replacing FM with plant protein sources (soybean and sunflower meal), except for FCR, which was the best in group D4 (1.90); approximately 6% higher than that of the control group (15% FM) (*p* = 0.04). Moreover, the VSI, HSI, and RGL were not affected by partial or total replacement of FM with plant protein (Table 2).

### 3.2. Hematological and Biochemical Indices and Fish Body Composition

Despite there being no significant difference among the experimental groups, the D4 and RD groups showed approximately similar results for the examined hematologic parameters (Table 3). The GH level was numerically decreased in the D4 compared to RD group, but the difference was not significant (*p* = 0.09) (Table 3). Gradually replacing FM with plant protein did not show any significant differences among the different experimental groups except for serum lipase enzyme (*p* = 0.000), which was the highest in the RD group (19.66 U/L), but the lowest in the D2 (9.33 U/L) and D4 (8.66 U/L) groups (Table 3). The serum liver and kidney function test showed no significant differences among the experimental groups in serum AST (*p* = 0.93) and ALT (*p* = 0.51), creatinine (*p* = 0.34), urea (*p* = 0.48), and BUN (*p* = 0.48) (Table 3). Regarding body composition (crude protein, lipid, moisture, and ash), the fish group fed a diet supplemented with 10% sunflowers meal (D4) showed numerically better values than the other groups, but the difference was not significant (Table 4).

### 3.3. Photomicrograph of Cross-Sections of Fish Intestinal Parts and Liver Histology

Histomorphology examination of sections of fish intestinal anterior parts showed a free lumen with intact separated tall villi with normal mucosa in the RD group. Furthermore, branched (increase absorptive surfaces), tall, and separated villi were noticed in the D1 group, while tall and thin villi with a narrowed lumen were observed in the D2 and D3 groups. Additionally, a free lumen with tall adhesion villi was seen in the D4 group. The middle parts showed a lumen with undigested feed elements followed by short and separated villi with normal submucosa in the RD and D1 groups. Furthermore, a lumen with many undigested feed elements with marked fusion and short villi were noted in the D2 group, while a free lumen with nearly tall, separated villi with marked widening of lacteal vessels was noticed in the D3 group. A few feed parts in the lumen with numerous fusion villi adjacent to tall separated and serrated surfaces villi were seen in the D4 group.

**Figure 1:**
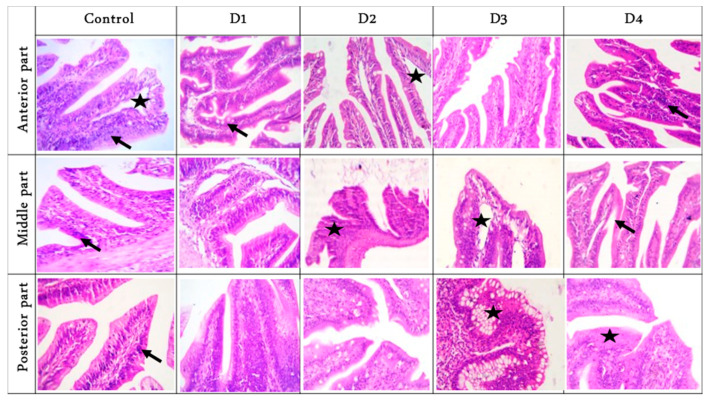
Representative photomicrograph of the cross Hematoxylin and eosin (H&E) stained sections of fish intestinal parts (anterior, middle, and posterior), magnification ×400, showed: Anterior parts: separated villi with prominent lacteal (star) beside sub-mucosal limited lymphocytic infiltration (arrow) in the control group. More branched (increase absorptive surfaces) with mild goblet cells in D1 group. Tall, thin villi with widening lacteal (star) were noticed in D2. Serrated sides (increase absorptive surfaces) were seen in D3. Adhesion villi (star) were seen in the D4 group; Middle parts: short and thick villi due to increased enterocytes admixed with lymphocytes (arrow) in both control and D1. Lumen with undigested feed elements with marked fusion and short villi (arrow) in D2. Free lumen with marked widening of lacteal (star) in D3. Irregular and thin villi surface due to a loss of enterocytes (arrow) were seen in the D4 group; Posterior parts: thin separated villi with pointed tips and infiltration of sub-mucosal lymphocytes (arrow) in the control group. Marked fusion villus due to hyperplasia enterocytes was seen in D1. Broad villi tips (increase absorptive surface) with narrowing lumen were noticed in D2. Marked goblet cells hyperplasia (star) with short villi was observed in D3. Short villi with increase enterocytes with near broad tips (star) were prominent in D4. D1, D2, D3, and D4: Diets were composed of 10, 7.5, 5, and 0% fishmeal, respectively.

**Figure 2:**
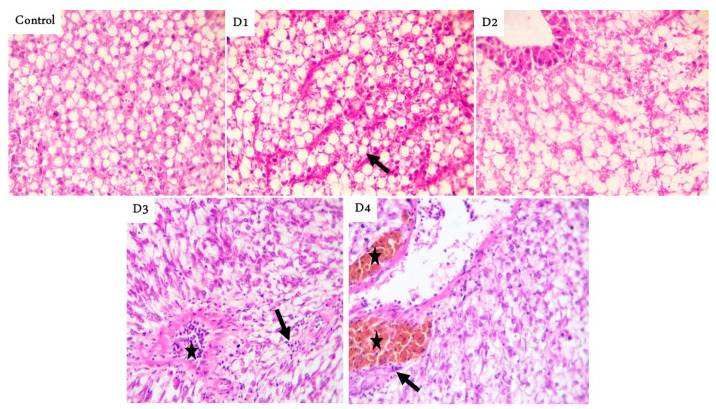
Representative photomicrograph of Hematoxylin and eosin (H&E) stained fish liver, magnification ×400 showing: normal hepatic parenchyma with fatty cytoplasm (arrow) decreases gradually in control, D1, and D2. Normal hepatocytes with prominent blood vessels contain lymphocytes in their lumen (star) and slightly infiltrated surrounding hepatic parenchyma (arrow) in D3. Prominent perivascular dark-brown melano-macrophages (stars) and slight lymphocytic infiltrations in the neighboring hepatic parenchyma followed with normal hepatocytes in the D4 group. D1, D2, D3, and D4: Diet was composed of 10, 7.5, 5, and 0% fishmeal, respectively.

The posterior parts showed tall branched and fusion villi beside a few cup-shaped villi tips with mild thickening of the muscular coat in the RD group. Moreover, marked fusion villi with a few feeds in the lumen were seen in the D1 group, while tall and branched villi with a narrowing lumen were noticed in the D2 group. Marked thickening of the muscular layer with short villi were observed in the D3 group. Finally, broad tips (a good mark of increased absorptive surfaces) were prominent in the D4 group (Figure 1). Histologic examination of liver sections showed normal hepatic parenchyma in the RD, D2, D3, and D4 groups, with notorious multifocal dark-brown melano-macrophages in the D1 group (Figure 2).

### 3.4. Myostatin Gene Expression

**Figure 3:**
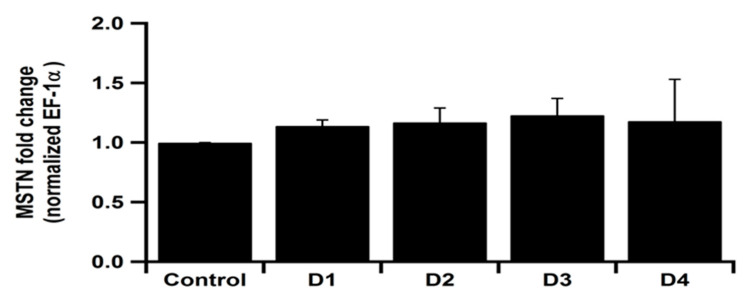
Myostatin (*MSTN*) gene expression in spleen of *Clarias gariepinus* fed experimental diets. Control diet was composed of 15% fishmeal as the main source of protein. D1: Diet that partially replaced fish meal with soybean meal (10% fishmeal). D2: the fishmeal was totally replaced with soybean meal. D3: the diet was supplemented with 10% sunflower meal with soybean meal and 0% fishmeal. D4: the diet was supplemented with 20% sunflower meal with soybean meal and 0% fishmeal.

Myostatin gene expression did not reveal any significant differences among the experimental groups (Figure 3).

### 3.5. Economic Efficacy

Gradually replacing FM with plant protein had a highly significant effect on the economic efficiency. The 15% FM diet (RD) was the most expensive diet, while D4 was the least expensive. D4 showed the best profit and economic efficiency compared to the other groups: 71%, 45%, 34%, and 21% better profit compared to RD, D1, D2, and D3, respectively. Moreover, the economic efficiency was greater in the D4 group compared to the RD, D1, D2, and D3 groups (83%, 62%, 52%, and 35%, respectively; Table 5).

## 4. Discussion

Our study of fish fed a diet completely replacing FM with soybean and sunflower meal revealed a similar growth performance as a diet containing 15% FM. Partial substitution of FM with plant protein has been achieved in various carnivorous fish [39,40], rainbow trout *Oncorhynchus mykiss* [41] and *Tilapia rendalli*, *Coptodon rendalli* [21,42], while complete substitution was successful in a small number of studies [43,44]. This may be because of a variety of factors, including: the component of the feeds used, culture technique, fish species, different aptitude of fish to consume plant protein, and the fact that most studies are performed in laboratory-based conditions [21,45,46].

There have been conflicting results on the impact of partial or total replacement of FM with plant protein in a fish diet. While several researchers have reported no difference [21,22,47,48,49], others have detected that this replacement caused poor growth performance [50]. Nevertheless, the current results agreed with the preponderance of research showing that a similar growth performance between groups fed FM and a diet totally replaced with sunflower meal, and this was assured by the unchanged level of serum GH among different groups. Replacing FM in the diet of *Tilapia rendalli*, *Coptodon rendalli* with diets including 10% and 20% sunflower protein [21], up to 25% of sunflower meal in diets of Mozambique tilapia *Oreochromis mossambicus* [47], and 65% sunflower meal in diets of rainbow trout [49] provided comparable growth performance in laboratory circumstances. These results were supported and compatible with our current findings.

Different plant proteins, for instance soybean meal, rapeseed meal, cottonseed, sunflower seed, lupin seed, and pea seed, are inadequate in ≥1 essential amino acid, especially lysine and methionine, which are requisite for fish feeding [51]. Moreover, the different alternative protein supplies may influence the enzyme activities, which are concerned with protein metabolism [52]. However, this is not the case in the current study, and this finding may be attributable to adding essential amino acids (methionine and lysine), and the fact that total protein ranged from 27.8% to 37.4% [53]. Furthermore, experiments on the palatability, digestibility, and feed consumption of sunflower cake in tiger shrimp, *Penaeus monodon* diet revealed encouraging outcomes [54]. The current performance traits were in accordance with Goda et al. [55], who stated that SBM can completely replace FM in diet for African catfish without any hazard effect on growth performance, especially if they are reared in earthen ponds and fed daily to satiation [56]. This may be based on the better nutritive level, amino acids availability and digestibility of SBM. The digestibility of soybean meal crude protein is higher than 90% [57]. SBM is considered the best source of protein for channel catfish with a promising amino acids profile [17]. Carnivorous blue catfish *Ictalurus furcatus* reared well on feeds with 70% of crude protein from SBM [58].

The results of this study regarding feed conversion ratio were the best in the D4 group. It was lower than the reported values of others [59], while it was compatible with the findings recorded in *C. gariepinus* in other studies [60,61,62]. On the other hand, some researchers detected higher FCR when completely replacing FM with sunflower meal, which was attributable to the amino acid balance and enhanced fatty acid contents in FM [21,63,64,65]. Lipid metabolism occurred mainly in the liver; therefore, food imperfections may be detrimental and trigger disorders in liver function, which is noticeable as liver fattening with high HSI estimates. This experiment revealed no significant variations in the HSI and VSI indexes among the different experimental groups. This was supported by Rocha et al. [66], who reported that the non-significant shifts in HSI among the different experimental groups denoted the appropriateness of sunflower meal for lipid metabolism [66].

Hematologic components of blood are valuable when monitoring feed toxicity, especially with feed constituents that affect the formation of blood [67]. Osuigwe et al. [68] reported that fish hematological indices are influenced by a variety of issue that embraces fish mass, age, physiological condition, environmental situations, and feeding procedure (i.e., components quality and quantity, protein sources, vitamins, and probiotics). The results of this study for hematological indices were similar to the values detected by others [60,61,62,69]. Moreover, the hematological parameters assessed in the current experiment were within the physiological ranges for *C. gariepinus* that were supported by Kumar et al. [70] and Rahmdel, Noveirian, Falahatkar, and Lashkan [25], who stated that there were no significant variances in hemoglobin, hematocrit, MCV, MCH, and MCHC values in common carp when replacing FM with kernel meal and sunflower meal, respectively. These results suggested that replacing FM with plant protein sources had no harmful consequence on blood parameters, since plant components decrease hematocrit level, which is considered a hazard for the wellbeing of farmed fish [71]. The high values of these parameters may be signs of disorders in liver, spleen, blood toxicity, or anemia [72]. Consequently, the comparable levels of these parameters in all our experimental groups showed that the hematopoietic tissues were performing normally.

High serum enzymes activities (AST and ALT) in wild *C. gariepinus* may be attributable to destruction of the hepatic cell [73]. These serum enzymes are cytoplasmic in nature and are only liberated into the circulation after these cells have been damaged [74,75,76,77]. With there being no difference among the experimental groups in our study, our results regarding AST (<35 U/L-1) and ALT (<41 U/L-1) were within the normal range. Consequently, these results revealed normal liver function in fish of the investigated groups. Replacing FM with plant protein sources revealed an increase in goblet cells that secrete mucus with an increase in muscularis thickness. These results may lead to increased mucus secretion and absorptive surfaces [62]. In our study, gradual replacement of FM had no effect on serum amylase enzyme activity; however, unexpectedly, lipase enzyme activity was markedly decreased in the D2 and D4 groups, indicating alteration in lipid digestion, where digestive enzymes provide information about the digestive capacity of fish to hydrolyze feed ingredients such as carbohydrate and lipid [78]. The negative impacts of D2 and D4 on lipase activity may be attributed to the various anti-nutritional factors, such as protease inhibitors, that are present at high levels in soybean and may reduce the activity of digestive enzymes in fish [79]. Many factors affect the digestive enzymes secretion in fish, including feed preferences and feeding habits [80,81]. Significant reduction in lipase and amylase activity in intestinal content of red seabream *Pagrus major* fed a soybean meal diet has been reported [82]. Similar findings also have been demonstrated in Atlantic cod *Gadus morhua* [78], Atlantic salmon *Salmo salar* L. [83], and hybrid tilapia *Oreochromis niloticus* × *O. aureus* [84].

Results were conflicting regarding the fish body composition because of replacing FM with plant protein sources in a fish diet. This may be due to various factors, including culture conditions, feed composition, environment, fish size, and genetic traits [85], but where fish body composition varies considerably from one fish to another in the same species in addition to within an individual fish [2], the feed composition is the chief factor that affects fish body composition [86]. Results have been conflicting on the effect of replacing FM with sunflower meal on fish body composition. While some researchers have detected no difference [22,87], others have reported decreased fish body fat and protein content with replacement of up to 20% of sunflower meal [21]. However, our results were comparable to the findings of the majority stating that the protein ranged from 54.23% to 59.40% [60,61,62]. Moreover, the fat contents and ash were similar to other results [22,48,62,87] when replacing fishmeal with sunflower meal.

Intestinal morphology modifications that were caused by plant protein were a crucial in the assessment of the potential value of an ingredient in fish diet. Additionally, the length of villi is considered a valuable histological factor that can be checked in the fishmeal replacement experiments and the assessment of different commercial diet. There was a positive relationship between nutrient digestibility and submucosal thickness of the intestine [88], with better protein, lipid, and energy digestibility. This may be attributed to this thickness increased the absorption surface area.

The mixture of plant protein improved the intestinal submucosal thickness without impairment of feed conversion [89]. These results confirmed our findings especially for D4. Moreover, there are no signs of enteritis caused by feeding soybean meal especially in European sea bass [88,90]. These outcomes supported the current results regarding the intestinal histological examination of catfish fed on plant protein, which may be attributed to; (a) less sensitivity of catfish to soybean anti-nutritional factors, similar to the European sea bass [88,90]; (b) low levels of saponin in soybean (5 and 7 g kg^−1^); (c) very low or absence of saponin in sunflower meal [12,91]. Aanyu et al. [92] and Couto et al. [93] confirmed our results that sunflower cake did not affect intestinal fold length and numbers. On the contrary, soybean caused histological, pathological, and functional alterations (subacute enteritis) in the gastrointestinal tracts of several fish species, particularly in salmonids [94], which may be due to synergistic effects of several antinutritional factors [95].

Fish have a distinctive position among vertebrates. They are inclined to grow indeterminately during life, providing the majority of this growth to build up muscle tissue [96]. Post-larval muscle growth in fish leads to enhanced hypertrophy and hyperplasia. However, in mammals, hyperplasia stops shortly after embryonic development and additional muscle development is caused chiefly by hypertrophy of the present fibers [97]. *MSTN* is one of the transforming growth factor-β superfamily that inhibits the growth and regulation of skeletal muscle mass [98,99]. Regardless of the dissimilarities among fish and mammals in *MSTN* expression imitates and myogenesis, recent research using transgenic zebrafish and medaka with a deficient signaling pathway for *MSTN* sustain the idea that *MSTN* prevents muscle development even in fish [100,101,102,103]. However, the *MSTN* in the current study was comparable among the experimental groups, which reflects that replacing fishmeal with plant protein (sunflower and soybean meal) did not affect the *MSTN* and growth in catfish.

The cost–benefit analyses revealed that FM replacements are economically attractive, with the prospective of replacing plant protein with animal protein sources being established to be of competitive nutritional value with reasonably lower costs. Our results showed that replacing FM with plant protein sources reduced the feed cost and improved the profit and economic efficiency. This was supported by Dayal, Rajaram, Ambasankar, and Ali [54], who stated that 20% substitution of fish with sunflower seed meal may decrease the feed cost (more than US$0.015/kg of fish produced). Moreover, Olvera-Novoa, Olivera-Castillo, and Martínez-Palacios [21] detected that the feeding cost was decreased for diets consisting of 10 and 20% sunflower meals concurrently with comparable performance to the control diets of *Tilapia rendalli*, *Coptodon rendalli*. Although sunflower seed meal is available, the importance of their involvement as replacement for FM has not been investigated effectively in earthen pond conditions, which are prevalent in developing countries [104]. Therefore, further exploration is needed to reveal whether this replacement will achieve the same experimental findings that would further improve the economic efficiency.

## 5. Conclusions

Fish and fish products have an imperative role in food security and the nutritional needs of the human population. Consequently, partial or total replacement of FM with a plant protein source showed a comparable growth performance and body composition with greater economic efficiency. Therefore, it is recommended to encourage fish producers to substitute the fishmeal with the soybean and sunflower meal when rearing and producing catfish, but with the addition of synthetic amino acids.

## Figures and Tables

**Table 1:** Ingredient composition and calculated nutrient content values of the experimental diets (% Dry matter).

Ingredients	Experimental Diets (g/kg) *				
	Control	D1	D2	D3	D4
Fish meal 60%	150	100	75	50	0
Soybean meal 48%	411	453	475	509	500
Sunflower meal 36%	0	50	71	81	100
Wheat bran 14.5%	128	68	38	0	0
Ground yellow corn	286	303	311	323	303
Corn gluten 60%	0	0	0	0	50
Fish oil	20.0	20.7	22.4	24.5	25
Dicalcium phosphate	0	0	2	6.6	15
Vitamin-mineral premix **	3	3	3	3	3
Dl-Methionine	2	2.3	2.3	2.5	2.7
L-Lysine	0	0	0.3	0.4	1.3
Calculated composition (% DM)					
Crude protein	32.2	31.9	32.2	32.1	32.2
Crude fiber	3.75	3.95	3.98	4.05	4.12
Starch	21.1	21.0	20.8	21.0	20.9
Ether extract	5.35	5.11	5.13	5.14	5.12
Lysine	1.89	1.88	1.86	1.89	1.87
Methionine	0.80	0.80	0.79	0.78	0.79
Cysteine	0.42	0.41	0.43	0.43	0.42
Threonine	1.23	1.24	1.24	1.25	1.26
Arginine	2.26	2.29	2.30	2.30	2.28
Histidine	0.74	0.78	0.80	0.82	0.85
Isoleucine	1.33	1.40	1.43	1.45	1.49
Leucine	2.43	2.52	2.56	2.61	2.96
Phenylalanine	1.44	1.55	1.59	1.64	1.77
Tyrosine	1.09	1.10	1.11	1.13	1.21
Tryptophan	0.39	0.42	0.43	0.44	0.45
Valine	1.60	1.69	1.73	1.76	1.82
Calcium ***	0.99	0.77	0.69	0.68	0.63
Available P ***	0.53	0.39	0.35	0.35	0.35
DE *** (kcal/kg diet)	2660	2656	2658	2659	2662

* Control diet composed 15% fishmeal. D1, D2, D3, and D4 were composed of 10%, 7.5%, 5%, and 0% FM, respectively ** Vitamin-mineral premix kg ^−1^ diet: Vit. A 8050 IU, Vit. D3 2100 IU, Vit. E 300 mg, Vit. k3 14 mg, Vit. C. 294 mg, Vit. B1 19.6 mg, Vit. B2 30.1 mg, Vit. B6 14.7 mg, Vit. B12 0.02 mg, Ca-D-Biotin 0.2 mg, Folicacid 0.4 mg, cholinHcl 1.0 g inosit. 3000.0 mg, pantothemic acid 50.0 mg, Nicotinic acid 100 mg, P-Aminobenzonic acid 50.0 mg. Minerals mix.: Each kg contain manganese 60 g, iron 80 g, copper 5 g, zinc 40 g, selenium 0.15, and iodine 0.35 g. *** All composition was calculated according to NRC [26]. DE (Digestible energy) was calculated by applying the coefficient of 0.75 to convert gross energy to digestible energy [27].

**Table 2:** Growth performance and biometric indices of *C. gariepinus* fed different experimental diets.

Parameters	Control	D1	D2	D3	D4	Pooled SEM	*p*-Value
IBW (g)	50.28	50.56	50.90	50.55	50.66	0.34	0.22
FBW (g)	108.29	107.84	107.02	107.94	107.37	0.14	0.09
WG (g)	58.01	57.28	56.12	57.39	56.71	0.36	0.51
SGR (%)	1.28	1.27	1.24	1.26	1.26	0.005	0.34
FCR	2.02 ^a^	1.99 ^ab^	2.0 ^a^	1.94 ^ab^	1.90 ^b^	0.002	0.04
VSI (%)	5.59	6.39	4.72	5.25	5.20	0.20	0.93
HSI (%)	1.41	1.15	1.30	1.01	1.07	0.11	0.33
Intestinal body ratio	0.95	0.80	0.92	0.93	0.97	0.06	0.15

Control diet was composed of 15% fishmeal. D1, D2, D3, and D4 were composed of 10%, 7.5%, 5%, and 0% FM, respectively. IBW: initial body weight. FBW: final body weight. WG: weight gain. SGR: specific growth rate. FCR: feed conversion ratio. VSI: Viscera somatic index. HSI: Hepatosomatic index. RGL: Relative gut length. The values with different superscripts (^a,b^) within the same rows were significantly different (*p* < 0.05).

**Table 3:** Hematological and biochemical indices, growth hormone levels, and digestive enzymes of *C. gariepinus* fed different experimental diets.

Parameters	Control	D1	D2	D3	D4	SEM	*p*-Value
RBCs (10^6^ µL^−1^)	1.70	1.69	1.32	1.33	1.57	0.07	0.21
Hct (%)	17.90	18.73	13.03	14.40	16.70	0.77	0.75
Hb (g dL^−1^)	6.06	6.06	4.53	4.46	5.60	0.27	0.14
MCV (fl)	110.90	105.60	98.63	107.70	107.90	1.70	0.20
MCH (pg)	35.86	35.56	34.36	33.60	35.26	0.39	0.35
MCHC (g dL^−1^)	33.96	32.13	34.96	31.23	32.89	0.58	0.28
ALT (UL^−1^)	23.56	24.83	26.36	25.2	22.03	0.78	0.51
AST (UL^−1^)	35.93	33.20	33.86	33.16	34.13	1.01	0.93
Urea (mg dL^−1^)	31.60	30.46	27.43	30.76	26.16	1.07	0.48
Creatinine (mg dl^−1^)	0.79	0.74	0.77	0.79	0.80	0.01	0.34
BUN (mg dl^−1^)	14.74	14.21	12.80	14.35	12.21	0.50	0.48
GH (pg mL^−1^)	529.53	543.40	499.70	500.87	495.23	16.63	0.099
Lipase (U L^−1^)	19.66 ^a^	19.00 ^a^	9.33 ^b^	16.00 ^a^	8.66 ^b^	1.32	0.0001
Amylase (U L^−1^)	23.00	22.66	23.66	21.33	22.03	1.30	0.55

Control diet was composed of 15% fishmeal. D1, D2, D3, and D4 were composed of 10%, 7.5%, 5%, and 0% FM, respectively. SEM: standard error of the mean, RBCs: Red blood cells. Hct: The hematocrit. Hb: Hemoglobin. MCV: Mean corpuscular volume. MCHC: Mean corpuscular hemoglobin concentration. ALT: Alanine Aminotransferase, AST: Aspartate Aminotransferase, BUN: Blood Urea Nitrogen, GH: growth hormone. The values with different superscripts (^a,b^) within the same rows were significantly different (*p* < 0.05).

**Table 4:** Nutritional composition of whole-body *C. gariepinus* fed different experimental diets (% on dry weight basis).

Parameters	Control	D1	D2	D3	D4	SEM	*p*-Value
Crude protein	57.70	57.33	57.30	56.96	58.03	0.66	0.99
Crude fat	13.33	13.00	17.66	13.33	14.66	0.71	0.20
Ash	22.40	24.93	21.50	25.40	23.06	0.74	0.45

Control diet was composed of 15% fishmeal. D1, D2, D3, and D4 were composed of 10%, 7.5%, 5%, and 0% FM, respectively. SEM: standard error of the mean.

**Table 5:** Economic efficiency of the different experimental diets.

Parameters	Control	D1	D2	D3	D4	SEM	*p*-Value
Feed $/kg BWG	1.28 ^a^	1.08 ^b^	1.00 ^b^	0.90 ^c^	0.74 ^d^	0.05	<0.000
Profit/kg weight gain $	0.22 ^e^	0.42 ^d^	0.50 ^c^	0.60 ^b^	0.76 ^a^	0.05	<0.000
Economic efficiency	0.18 ^e^	0.39 ^d^	0.50 ^c^	0.67 ^b^	1.03 ^a^	0.08	<0.000

Control diet was composed of 15% fishmeal. D1, D2, D3, and D4 were composed of 10%, 7.5%, 5%, and 0% FM, respectively; $/kg diet = $0.63 for control diet (15% fishmeal); $0.54 for D1 (10% fishmeal); $0.50 for D2 (7.5% fishmeal); $0.46 for D3 (5% fishmeal); $0.39 for D4 (zero fishmeal), SEM: standard error of the mean, BWG: body weight gain. The values with different superscripts (^a–e^) within the same rows were significantly different (*p* < 0.05).

## Data Availability

All data sets collected and analyzed during the current study are available from the corresponding author on fair request.
